# Exploring waste disposal attitudes and market strategies for recycled construction and demolition waste in India

**DOI:** 10.1038/s41598-025-32996-7

**Published:** 2025-12-29

**Authors:** Hammadhu HaitherAli, Salman Shooshtarian, Anjali Gopakumar

**Affiliations:** 1 School of Civil Engineering, Vellore Institute of Technology, Chennai, 600127 India; 2 School of Property, Construction and Project Management, RMIT University, Melbourne, Australia

**Keywords:** Circular economy, Stakeholder, Market analysis, Construction and demolition waste, Secondary material, Engineering, Environmental sciences, Environmental social sciences, Environmental studies

## Abstract

The improper disposal of construction and demolition waste (CDW) poses significant environmental and infrastructural challenges in India. This study investigates the barriers and opportunities associated with promoting the use of recycled secondary materials (SM). A mixed-methods approach was adopted, combining quantitative analysis of closed-ended survey responses with thematic analysis of open-ended responses. Data were collected from 73 stakeholders in Chennai, and findings were validated through triangulation. Results indicate low awareness of local waste management regulations (14%) and non-compliant waste disposal practices (> 60% disposing on roadsides, waterbodies and open lands). Quality emerged as the most critical factor influencing the purchase of SM (14.11/15), while reluctance to adopt (85% not willing) stemmed from concerns over procurement distance, costs, and the absence of client demand. Qualitative findings further reinforced quality concerns as the dominant barrier. To address these challenges, the study highlights the need for strong policy support (fiscal incentives), targeted marketing strategies (decentralised supply, diversified products), and improved industrial readiness (stakeholder awareness). Overall, the research identifies key factors enabling circular transition by emphasising consumer insights for SM market development and revealing non-compliant behaviours. Findings align with SDGs (9,11,12) and provide actionable insights for policymakers and recycling businesses.

## Introduction

Rapid urbanisation and economic development have resulted in higher demands for residential buildings and infrastructure, leading to a significant increase in construction and demolition waste (CDW) generation^1,2^. Globally, more than ten billion tons of CDW are produced annually, accounting for 35% to 65% of landfill space^3^. Advanced economies, such as Austria, Finland, the Netherlands, and Germany, have effectively managed to redirect waste from landfills. However, despite the potential for recovery and recycling, over 90% of CDW is disposed of in dumpsites or waterbodies in developing countries like India and China, leading to environmental issues^4–6^.

To prevent resource wastage and promote circularity, emerging economies such as India are advised to prioritise recycling, although not a preferred option, as it is more achievable than reduction and reuse options^7^. India alone generates over 100 million tonnes of construction waste and 300 million tonnes of demolition waste annually^8^. In response to this issue, the Government of India has enacted a policy (*Construction and Demolition Waste Management Rules*,* 2016*) to streamline CDW management and promote recycling, encouraging cities to install recycling facilities and ban illegal dumping^9^. Despite the enforcement of this regulation, many cities across the country have not been able to develop recycling infrastructure and continue to practice open waste dumping^10,11^. Enforcement is complicated due to the involvement of various stakeholders, including governments, contractors and recycling facilities in the systems^12^. Unfortunately, the interests of these stakeholders differ from one another^13^. Hence, it is argued that understanding the perspectives of regional stakeholders is crucial for the effectiveness of waste management systems, particularly in developing nations, where CDW management is often perceived as the responsibility of the government. By actively engaging stakeholders, policymakers can address concerns, foster initiative acceptance, and gain insights into market needs^12^.

Additionally, engaging stakeholders helps ensure relevance, identify and mitigate risks associated with policy reforms and potential changes in their enforcement^15^. Hence, an in-depth understanding of stakeholders is crucial to establish an effective waste management system that prevents resource wastage, promotes recycling and supports CE.

The SM market for recycled inert CDW is a complex domain influenced by various factors, including technological, environmental, economic, and policy considerations. Central to understanding this market is the role of stakeholders, whose behaviours, incentives, and interactions significantly shape the development and sustainability of recycled material flows. Existing studies from Australia^16–18^, China^19^, the UAE^20^, and Europe^21^ provide insights into these dynamics, emphasizing the importance of stakeholder analysis in fostering an efficient SM market. However, such studies are unavailable in India.

The existing literature on India analyses waste-generating factors^22–24^, environmental and economic assessment of CDWM^11,25–28^, barriers to sustainable CDWM^29,30^, on-site CWM practices in large construction companies^31,32^ and reviews of broader sectoral challenges^2,6,8,33,34^. Most of this work focuses on regulatory, environmental, or process-oriented aspects of WM. However, in-depth empirical investigation of contractors’ perceptions towards purchasing SM in the Indian context is scarce.

Since consumers are the primary driving force and the core of any business, it is crucial to understand the needs and expectations of construction contractors. They play a significant role as both consumers and suppliers of materials in the CDW recycling ecosystem^35,36^. CDW management and SM adoption are ultimately shaped by behavioural intentions and stakeholder interactions. Hence, this study draws on stakeholder theory and behavioural perspectives aligned with the Theory of Planned Behaviour, acknowledging that attitudes, perceived control and norms critically influence material choices and compliance behaviours^31,32^. Positioned within this theoretical framing, the present study addresses a distinct yet underexplored gap by examining contractors’ willingness, expectations, and barriers related to purchasing SM, thereby complementing the predominantly technical and policy-oriented Indian CDW literature with much-needed market and behavioural insights.

The objectives of this study are:

1) To assess contractors’ awareness of and compliance with local regulations on CDW, particularly regarding the mandated delivery of inert debris to recycling facilities.

2) To explore market insights and consumer attitudes toward SM, including their purchasing intentions

Despite policy mandates, the implementation of CDW recycling remains limited due to low contractor compliance and weak market demand for recycled products. This study examines on-ground realities to inform effective policies, enhance CE strategies, and support recyclers and material dealers.

This research supports SDG 11 (Target 11.6), SDG 12 (Targets 12.5 & 12.7), and SDG 9 (Target 9.4), reinforcing its contribution to global sustainability goals and responsible practices in the construction sector.

## Literature review

### An overview of recycling and challenges in the secondary material market: global context

Recycling acts as a core principle of CE. The annual global consumption of concrete exceeds 30 billion tonnes^37^. Coarse aggregates, for instance, account for 60% to 67%, while fine aggregates such as sand comprise 33% to 40% of concrete production^38^. Hence, there is an urgent need to prevent the exploitation of natural resources through recycling and reuse in the built environment sector^39^. However, there are several challenges involved in selling the SM and achieving circular transition in the construction sector, as listed in Table 1.

**Table 1:** Key challenges identified for SM market development and establishment.

Challenge	Description	Country	Reference
Quality perception affects demand	Consumer perception influences willingness to buy SM	China	^40^
Price linked to perceived quality	In absence of quality info, consumers use price as a quality signal	China	^40^
Perceived value drives intention	Purchase decisions influenced by social, environmental, and economic values	China	^41^
Systemic regulatory & operational gaps	Lack of regulation, weak design focus, poor waste tracing, low coordination, and site sorting issues	China	^19,42,43^
Low client demand & poor infra	Lack of facilities, regulations, economic viability, quality, awareness, and stakeholder coordination	China	^44^
Higher cost than virgin material	Recycled concrete costs 0–10% more due to labour-intensive processes	China	^43^
Virgin aggregates dominate	Preference due to convenience, sourcing ease, and lower cost	Sweden	^45^
System-level barriers: market, policy, logistics	Barriers include weak governance, low awareness, poor infrastructure, and standard restrictions on uptake of recycled aggregates	Australia	^16,46^^18,47^,
Low awareness among companies	Firms unaware of quality, savings, or environmental benefits	USA	^48^
Low landfill fees, high transport costs	Lack of economic incentive to recycle; SM viewed as inferior	Australia	^49^
High energy & transport cost	Recycling costs exceed those of virgin material due to logistics and energy	UK	^50^
Compliance vs. economic concern	Regulations matter more to professionals; public influenced more by cost incentives	Hong Kong	^51^
Durability concern	Consumer & contractor doubt about durability and the ownership of quality, as demolition lies with informal sector	Developing nations	^52^
Stakeholder coordination	Contrasting objectives of stakeholders such as recyclers, regulatory authorities, consumers and waste generators	Review	^53^^54^,
Economic viability	Low profitability and limited market viability	Italy	^55^

### Regulatory and infrastructural landscape for recycling in India

In India, the national policy ‘*Construction and Demolition Waste Management Rule*,* 2016*’, prohibits the illegal disposal of CDW and mandates local authorities to install recycling facilities, designate disposal sites, fix penalties for illegal disposal and impose tipping fees. The policy further requires waste generators producing more than 20 tons per day or 300 tons per project to segregate waste into five categories: soil, steel, concrete, wood and plastics, and bricks and mortar. Furthermore, it mandates the storage of waste on the premises. It prohibits littering on public roads or drains, as well as actions that hinder traffic or public access^56^.

Despite policy mandates, enforcement remains weak, and municipalities are only gradually developing the necessary infrastructure for recycling. Demand for recycled aggregates and other inert products remains low, even in pioneer cities such as Delhi, which established recycling plants among the earliest in the country^57^. Limited plant capacity, gaps in technical expertise and fragmented regulatory frameworks further limit the market growth^30,58^. Notably, with the introduction of the new ‘Construction and Demolition Waste Management Rules, 2025’, which mandate Extended Producer Responsibility (EPR), there is scope for circular business models. It is now essential to assess waste generators’ awareness of these regulations and their disposal behaviours to build a robust waste diversion system. Without an effective waste diversion system in place to send waste to the recycling facility, it is difficult to establish the supply chain, as recycling enterprises are the primary suppliers of recycled materials.

## Methodology

### Research design

**Fig. 1:**
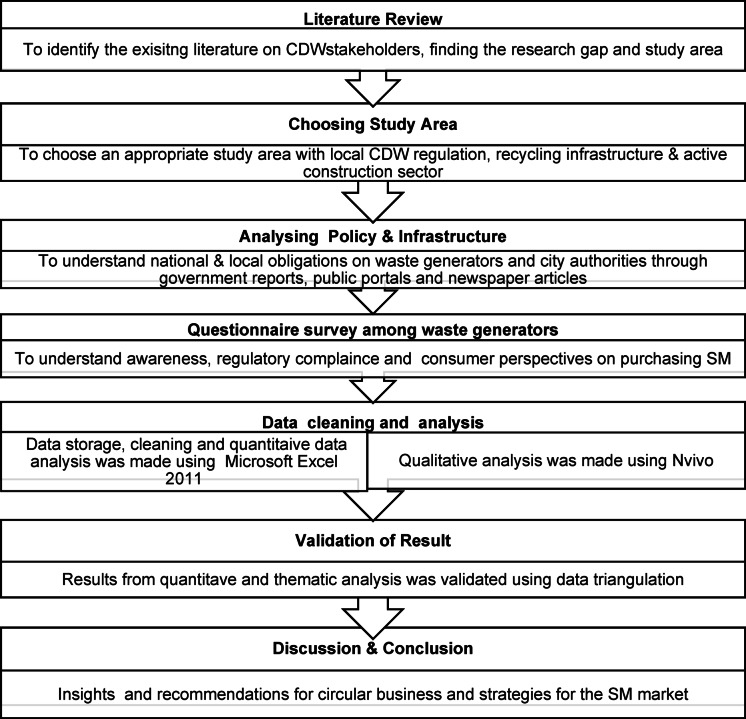
Outline of the research process.

This study employed an explanatory sequential mixed-method approach as outlined in Fig. 1. A questionnaire survey was distributed among CDW generators in Chennai, to gather comprehensive data on their perceptions, awareness and practices regarding waste disposal. Both quantitative and qualitative analyses were performed, and strategies for establishing a secondary market for inert recycled CDW were proposed in the end after validating results through triangulation. This approach provided a thorough understanding of on-the-ground realities, elucidating the reasons for policy failures, market demand for SM and the consumer insights on purchasing SM.

### Study area

Chennai, the fifth-largest megacity in India is chosen for the study. Spanning 1,189 sq. km and home to 12 million people^1^, Chennai is a major industrial and commercial hub with over 15,000 industries and a growing migrant population. The city is divided into 15 zones under the Greater Chennai Corporation (GCC), while urban planning is managed by the Chennai Metropolitan Development Authority (CMDA). Chennai generates 1,200–1,600 tonnes of CDW daily, accounting for 36% of its total solid waste, excluding illegal dumping^35^.

In adherence to the national policy, the city authority has installed two recycling facilities under the Public-Private Partnership (PPP) model and designated 15 places to dispose of CDW, one in each zone. Information about designated disposal sites is communicated through newspaper articles and the public portal. Private agencies manage the collection, transportation and recycling of waste in the city in collaboration with the corporation. The corporation imposes penalties ranging from INR 2,000 to 5,000 for illegal disposal^35^.

### Data collection

Data collection was performed through a structured survey, which comprised four sections: (1) Demographics, (2) Awareness and compliance with CDW regulations, (3) Willingness to purchase and expectations of SM, and (4) Challenges in procuring them. Survey questions were developed through a multi-stage process. First, an extensive review of existing literature on CDW management, recycled material adoption, local regulation, and secondary-material markets was conducted to identify relevant themes and construct the initial pool of questions^16,19,20,31,32^, 40–^55^. These themes were then aligned with the study objectives to ensure full coverage of regulatory awareness^56^, purchasing attitudes, market expectations, and perceived barriers. A mix of question types such as yes/no, Likert scale, multiple-choice, and open-ended questions, enabled the collection of diverse perspectives and actionable insights^59^.

Before finalising the questionnaire, a validation process was carried out to ensure clarity, relevance, and completeness. Two academic experts reviewed the content, followed by a pilot test with five respondents to identify issues related to wording, structure, or response options. Based on the feedback, unsuitable questions were removed, and others were simplified. The final version was printed in the local language for distribution.

All methods were performed in accordance with the relevant guidelines and regulations of the institution. The study protocol was reviewed and approved by the Institutional Ethical Committee for Studies on Human Subjects (IECH), Vellore Institute of Technology, Chennai [VIT/IECH/CC/2025/89]. Informed consent was obtained from all individual participants included in the study.

Participants were selected using the snowball and purposive sampling methods, based on the following criteria:

- Small and medium-scale building contractors in Chennai,
- Over five years of experience in residential construction and demolition.
- Decision-makers for material use and waste disposal in their projects.

Initial respondents were members of the local builders’ association. The survey was administered in person after obtaining verbal consent, with clarifications provided on-site to avoid misinterpretation. To encourage honest responses, particularly on sensitive issues like illegal practices, participants were assured of anonymity and the survey’s purpose in informing policy and improving infrastructure.

Each survey took 10–15 min. Responses were collected in hard copy, translated into English, and entered into Excel for analysis. The sampling method must align with the aims of the selected methodology; qualitative approaches prioritise depth and saturation, while quantitative approaches emphasise breadth^60^. A total of 73 responses were gathered through purposive and snowball sampling method. The sample size of 73 contractors is considered adequate for exploratory research in the Chennai city, where small- and medium-scale contractors are difficult to access and are reluctant to participate in regulatory compliance-related surveys. Data collection ended upon reaching saturation, where no new insights emerged from open-ended questions^61^.

Comparable studies utilise samples of < 25 to capture meaningful behavioural insights^62^, and methodological guidelines consider samples above 50 sufficient for exploratory stakeholder-perception research^60,63^.

### Data analysis and result validation

The entire data analysis, including descriptive statistics of demographic data, percentage and average calculations, and visualisations, was made using Microsoft Excel 2011. The survey included two types of scaled questions. For product preference (likelihood to buy), a standard 5-point Likert scale was used and analysed directly (1 = very likely to 5 = not likely) by calculating the mean score (Fig. 3b). For factors influencing the purchase of recycled materials (Fig. 3c), responses were categorised as High, Medium, or Low and converted into weighted scores using a linear scale (High = 15, Medium = 10, Low = 5). This weighting approach, commonly applied in construction-management studies, enables clearer differentiation between importance levels and allows qualitative judgments to be expressed as comparable numerical values for ranking and analysis^64^.

Formula used for calculating the mean score

$$\:\mathrm{M}\mathrm{e}\mathrm{a}\mathrm{n}\:\mathrm{S}\mathrm{c}\mathrm{o}\mathrm{r}\mathrm{e}\:=\frac{\varSigma\:{(w}_{i}\cdot\:{f}_{i})}{N}$$.

Where:

*w*_*i*_= weight assigned for each response, *f*_*i*_= frequency of response in each category and *N* = total number of respondents^64^.

The final open-ended question was grouped into categories based on themes using NVivo software, and the results were then validated through data triangulation. The qualitative data were coded using an inductive approach, which allows themes to evolve organically from real data and is appropriate for exploratory research^65^. The coding reliability was maintained through iterative review, memoing, and constant comparison across responses to ensure consistency in theme development. Triangulation was achieved by integrating the quantitative survey results with qualitative themes derived from inductive coding process^66^. Thus, triangulation ensured that the quantitative results were reinforced by the qualitative insights.

## Results

### Participants’ profile

The demographic details of the participants, including their education and experience, are summarised in Table 2. The responses were normally distributed across the categories.

**Table 2:** Summary of demographic details of the participants.

Respondent’s Profile Educational Qualification	Level of qualification	No. of respondents		
	Undergraduate	49		
	Diploma	16		
	Postgraduate	8		
*Experience*	Mean	Standard Deviation	Max No. of projects	Min No. of projects
	16 years	9 years	40	5
*Number of construction projects handled by the respondents*	21–30	11–20	100	2
*Number of demolition projects handled by respondents*	1–10	1–10	24	1

### Level of awareness about local CDW regulation and compliance (Objective 1)

**Fig. 2:**
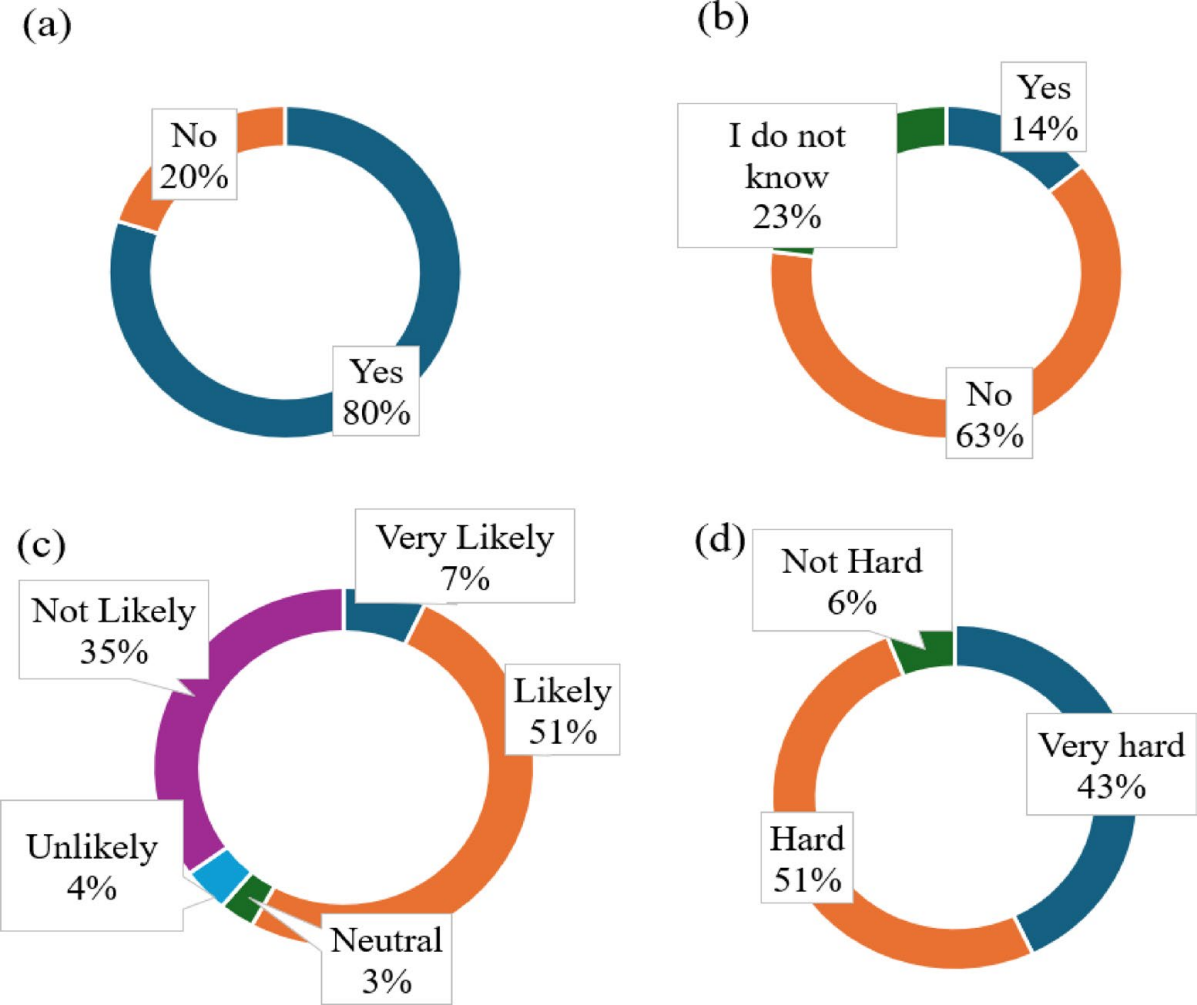
(**a**) Awareness of CDW recycling, (**b**) Awareness of local regulation on designated CDW disposal sites, (**c**) Willingness for onsite segregation, (**d**) Difficulty in onsite segregation.

Although the city has two recycling plants, only 20% of the participants were aware that CDW was being recycled (Fig. 2). The city authority has designated 15 specific sites for waste disposal; however, only 14% of the population is aware of this (Fig. 2b). The waste generators are unaware of local regulations and the legal ban on disposing of waste anywhere other than the designated disposal site. As the national policy mandates the onsite segregation of waste, the respondents were asked about their willingness to segregate CDW onsite (Fig. 2c) and the level of difficulty involved (Fig. 2d). The results indicate the unwillingness for onsite segregation.

On enquiring about how they dispose of the inert waste from their site. Depending upon the material and its demand, the disposal method varies as shown in Table 3 (a & b). The price for disposing of waste ranges between $6 and $60, equivalent, depending on the size of the vehicle, distance and area (rural/urban).

**Table 3:** (a)Various disposal methods followed for different CDW materials.

Material			Methods of waste disposal
Inert CDW (Purchased if there is a requirement for filling, and Waste is available from a single source in bulk quantity)	• Construction waste is left near the site if it is open or unoccupied land. • Given to waste removal vendors who dispose of at the site designated by the local authority, dumping yard, open places, or water bodies • Used for filling, backfilling, or raising the height of a low-lying area		
Steel (Has good salvage value)	• Sold to scrap dealers/vendors • The client will take and compromise on payment		
Wood (Has good salvage value)	• Wood is mostly reused onsite or diverted for reuse on other sites • Sold to vendor • Used for combustion if damaged beyond use or disposed of with other solid Waste		
Ceramic, Glass (No value)	• Disposed of other solid Waste		
Plastic & PVC (Has salvage value)	• Sold to a scrap dealer or vendor		
(b) Disposal of inert CDW material		% of respondents	
Dump on the roadside			3
Use for approach roads and ground levelling			6
Dump of waterbodies			10
Use on the same construction site			11
Report to the waste disposal service			25
Use in other construction projects			34
Dump on wasteland/unused site			59

From Table 3, it is evident that the inert CDW is disposed mainly of in inappropriate places such as open lands, roads and water bodies. Hence, there is low awareness and weak regulatory compliance.

### Insights into CE businesses and the SM market (objective 2)

According to the survey on purchasing point of new building materials (Table 4), contractors consistently prioritize suppliers based on reliability, proximity to the project site to minimize transportation costs, and suppliers serving as a single point of contact for most products. A contractor can find a building material supplier within a 2-kilometre stretch within the city. On the contrary, SM are only sold from the two recycling facilities in the city, which are far from most consumers. When the point of consumption is distant, significant transportation costs will be added to the material cost, making it uneconomical.

**Table 4:** Building materials procurement point.

Material	Point of procurement
Cement	Authorised local distributor Cement factory Local building material supplier
Coarse aggregate	Government-approved quarries Local building material supplier
Sand	Government-approved supplier Local building material supplier
Steel	Local building material supplier
Bricks and blocks	Brick Kiln Block manufacturing factories Local building material supplier

#### *Consumer preferences and attitudes towards purchasing SM*. In response to the question about the consumption of SM, it was found that most participants conveyed a lack of interest in procuring SM derived from CDW (Fig. 3a)

**Fig. 3:**
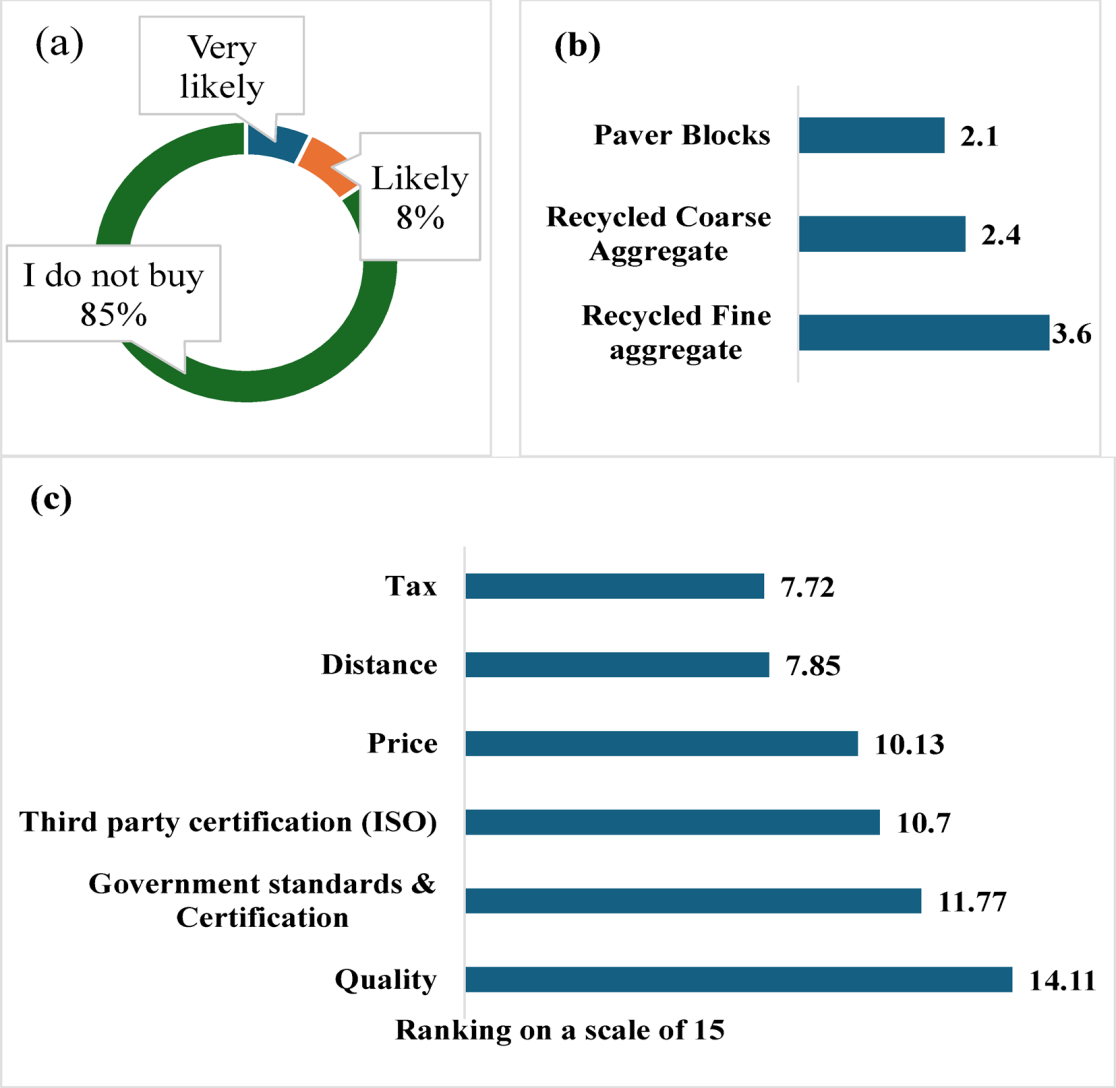
(**a**) Likelihood to buy recycled material, (**b**) Likelihood to purchase different SM on a scale of 5, (**c**) Ranking of factors influencing the decision to purchase SM on a scale of 15.

When consumers were asked to rank their likelihood of purchasing recycled products, the following responses were obtained: Sand, Coarse Aggregate, and Paver Blocks received first, second, and third rank, respectively (Fig. 3b). Hence, Fine aggregates, such as sand, have emerged as the most favoured option within the realm of recycled products. Delving deeper into the factors influencing the decision to purchase, consumers were asked to rank the importance of different factors they consider while purchasing SM using high, medium, and low categories. Numerical values were assigned to the rankings: High = 15, Medium = 10, Low = 5. The total scores for each factor were then calculated and ranked, with quality being the most crucial factor, as shown in Fig. 3c.

### Constraints to SM uptake and strategic interventions

The open-ended question, which inquired about challenges in purchasing SM for construction or any suggestions they would like to share regarding recycled CDW, was analysed using NVivo. The results were coded and categorised under themes (Table 5). The consumer prefers materials to be readily available within a 5-kilometre radius of the project site, underscoring the importance placed on just-in-time availability. Moreover, participants indicated a strong inclination towards sourcing all the required materials from a single reliable supplier to streamline their procurement processes efficiently. The respondents also highlighted that their purchase choice depends on client’s willingness, as client satisfaction is crucial for their business.

**Table 5:** Thematic analysis.

Category	Sub-theme	Count
Challenges in buying SM	Quality	23
	Price Concerns	6
	Client Willingness	6
Market Expectations in buying SM	Proximity & Distance	9
	Certification – ISO/ISI	5
	One-stop Supply	4
	Immediate Delivery	3
Regulatory Requirements for SM	Certification – Government	2
	Awareness creation	1
	Subsidy	10
	Tax Incentives	4

### Result validation

**Fig. 4:**
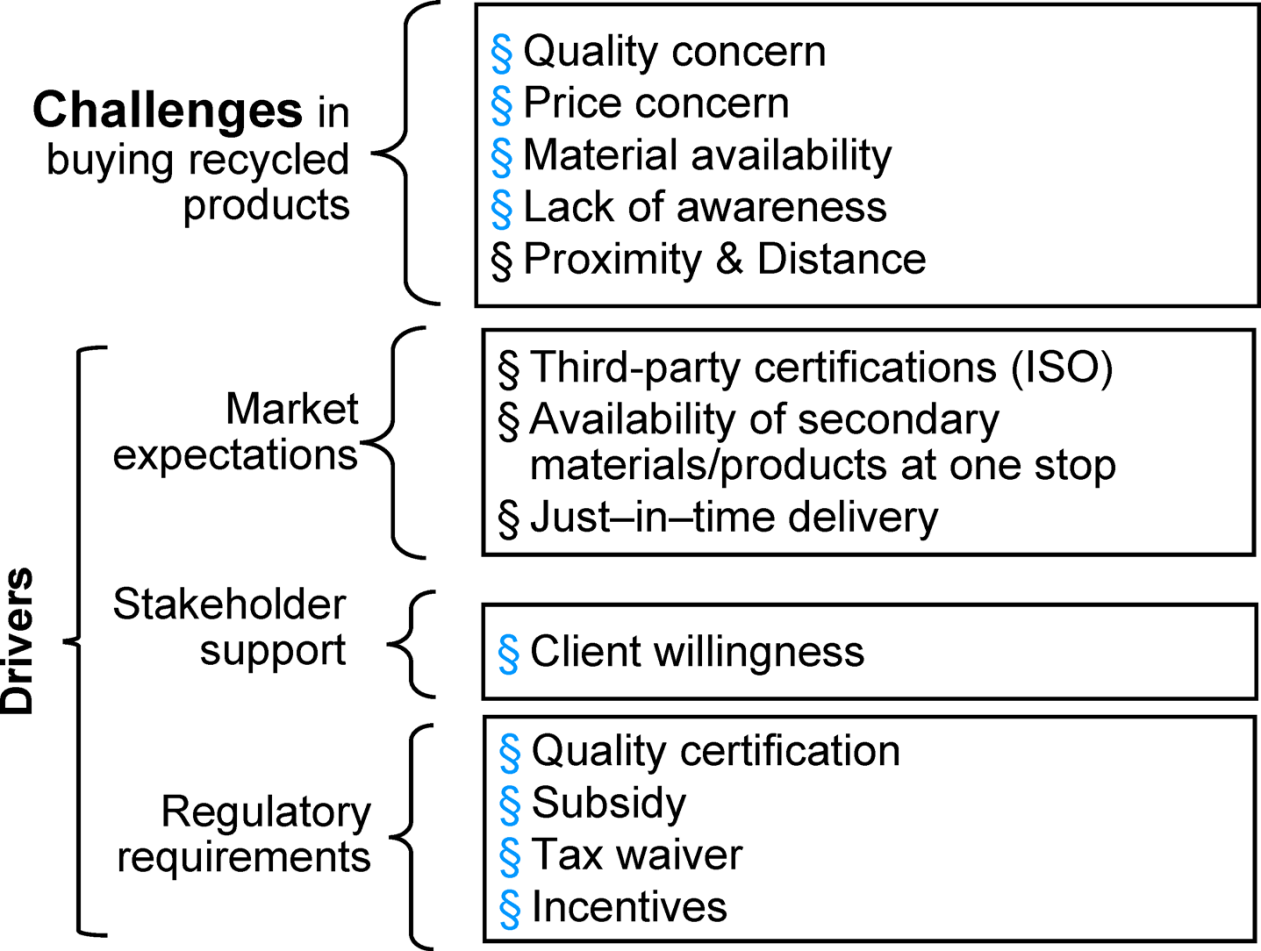
Challenges and drivers of the SM market for inert CDW.

Similar to the results in Fig. 3, findings from the thematic analysis also highlight that quality, procurement distance, and subsidy are important factors influencing the purchasing decision. Furthermore, client willingness emerged as a critical barrier in consuming SM. Taken together, this triangulated evidence demonstrates that technical factors (material quality), logistical challenges (supplier proximity), behavioural influences (client willingness), and policy instruments (subsidies and certification) jointly determine the market uptake of SM. The challenges and drivers of the secondary material market identified from the study are summarised in Fig. 4.

## Discussion

### Level of awareness and policy compliance

The results highlight the lack of awareness among waste generators regarding local regulations on waste disposal (4.1.2). This indicates a significant gap in the city’s communication and enforcement of waste management policies. Existing waste disposal methods underscore the prevalence of informal and environmentally harmful practices. Other than the waste used for backfilling, the rest remains undisposed of at the site or conveniently disposed of at a nearby open spaces or waterbodies^35^. Waste generators exhibit irresponsibility in their disposal choices, as it is influenced by ease and disposal costs. Several news reports have highlighted the issue, where even eco-sensitive marshland are being used as dumping grounds^67^.

Moreover, CDW is not segregated at the source despite the government’s rule (Fig. 2d). In addition to space constraints, committing time, money and labour to segregation is typically not in the interest of construction or demolition contractors. Such unsegregated disposal leads to poor production at recycling facilities. Several studies also reported that labour, time and cost constraints prevent demolishers from performing selective demolition^5,68,69^. The absence of a regional mandate for waste generation and diversion reporting creates loopholes for irresponsible waste disposal among waste generators. This highlights a regulatory gap that needs to be addressed to ensure proper waste management practices. A recent study conducted in Denmark also yields similar findings, where the lack of documentation on reclaimed materials, unclear responsibilities for end-of-life material management, and unwillingness to take the risk of buying or selling SM in new construction are reported as major inhibitors of CE^68^. Additionally, existing communication methods, such as conveying information through local newspaper articles about designated disposal sites, are ineffective in reaching waste generators. Hence, more compelling and accessible communication channels should be employed to disseminate crucial information to the stakeholders^70,71^.

The waste disposal practices depicted in Table 3 indicate the irresponsible behaviour of contractors. Contractors should demonstrate their dedication and commitment to environmental performance and waste reduction in the project by involving environmental experts, allocating resources, developing action plans for waste management, and selecting sustainable suppliers and materials^72^.

From inquiries on methods of disposal (Table 3), it is evident that materials with high salvage value, such as steel, wood, and plastics, have a significant secondary market and are not reaching landfills in substantial quantities. However, inert mineral parts, such as sand, stone, dead mortar, and other fines, have no market value. To avoid the expense of removing waste, they may leave it at the site or hire waste removal services, which dispose of it elsewhere at a lower cost. Studies in Australia, China, the UK and Denmark also highlighted that the cost of demolition and transportation prevents contractors from adopting CE practices^68,69,73,74^. Behavioural studies in India have also emphasised that individual attitude and institutional pressures play a crucial role in decision-making related to CDWM and on-site resource efficiency^31,32,75^.

### Key challenges in Establishing the SM market

This study identifies that lack of consumer awareness and interest in purchasing SM acts as a significant challenge in promoting the demand for recycled CDW products. Factors influencing purchasing decisions are found to be quality, adherence to standards and proximity, providing valuable insights for marketing and promotion strategies to increase the acceptance of SM. Notably, the tax for virgin aggregates is 5%, while the tax for recycled aggregates is 18%. Hence, targeted government interventions such as tax relaxation could promote the adoption of SM^16,19,46^. Countries such as Brazil, Hong Kong, and China have demonstrated that government interventions, including policies, taxes, and incentives, can help boost waste recycling^53,76–80^. Study show that mechanical properties of recycled aggregates, demonstrating that stakeholder acceptance depends on the quality and performance of recycled products^81^. These findings underscore the importance of stakeholder collaboration in research, quality assurance, and standards development to foster trust in recycled inert materials.

Lack of marketing is a significant cause of limited awareness and product visibility among consumers^35^. Hence, it is crucial to launch intensive targeted marketing to create awareness and improve the visibility of recycled products^58,82^. Diversifying products and developing codal provisions for utilising these products would promote sales^70,83^. Addressing logistical challenges and enhancing material availability and accessibility through improved recycling infrastructure are also crucial for overcoming the constraints in purchasing SM^52,58^.

Currently, SM are sold only through recycling factories. When asked about the point of material consumption for their project, all participants mentioned the nearest or known building material supplier. Hence, deploying existing distribution channels, such as local construction material vendors, to distribute SM would be an excellent strategy to boost sales due to supplier relationships, availability from nearby locations, and increased visibility^73,84^.

### Strategies to boost the secondary material market

**Fig. 5:**
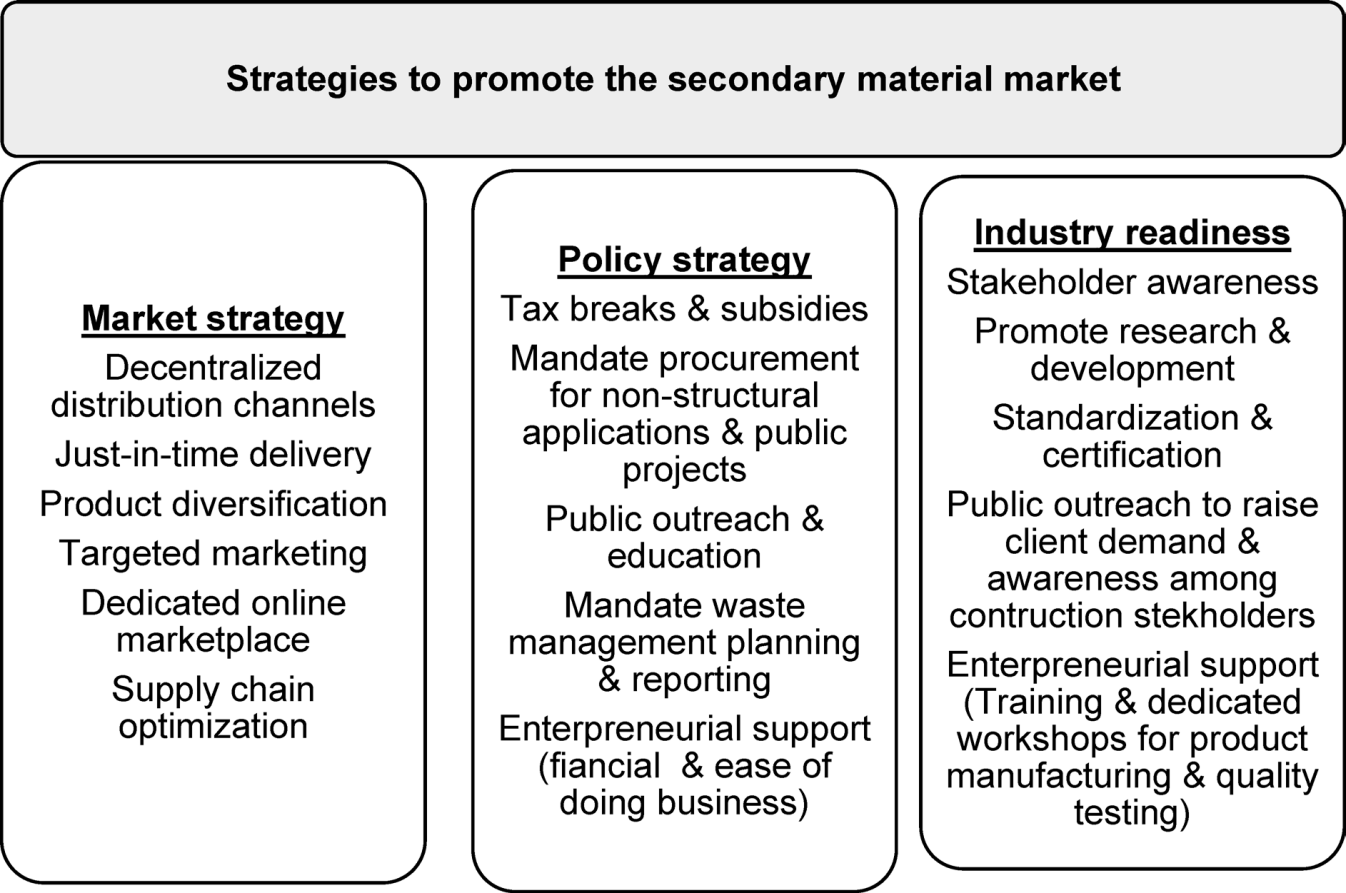
Strategies to promote the SM market.

In India, these challenges in recycling are compounded by informal labour practices and limited access to finance for small-scale recyclers. Since demolition is not standardised and there is an absence of clear responsibilities for the collection and reprocessing of materials, there is a need to push for selective demolition during the tendering process itself. Establishing standardisation and an online interactive platform connecting stakeholders in the material supply chain can provide opportunities for circular business models^68^. Targeted training programmes for recyclers and construction professionals, coupled with finance schemes to support small‐scale processing units, can boost the market competitiveness^85^. Certification by the government or a recognized international organisation can further enhance the reliability of the materials^15^. Finally, deploying technology in waste management systems and secondary market trading platforms will facilitate improved traceability, continuous improvement measures, monitoring and control. The strategies to achieve market robustness, successful policy and industrial readiness to promote a secondary market for inert CDW resources are proposed in Fig. 5.

There is a promising opportunity for recycling businesses, secondary market and CE integration due to the launch of the new policy ‘Construction and Demolition Waste Management Rule 2025’, which mandates EPR over waste generators and mandates the consumption of SM.

### Study’s implications for theory, practice and policy

This study offers important implications for both theory and practice, promoting the use of SM in construction. Theoretically, it advances our understanding of stakeholder behaviour by highlighting how client willingness, product quality, proximity, and certification influence purchase decisions, aligning with behavioural theories such as the TPB. It also contributes to CE frameworks by emphasizing the need for integrated policies, logistics, and market mechanisms to mainstream SM.

The *C&D Waste Management Rule 2025* introduces mandatory obligations requiring all construction projects to segregate waste at source and dispose of debris only at registered collection or recycling facilities. The Rule also mandates increased use of recycled products in construction works with target of 20% by 2030 and directs local authorities to enforce compliance through penalties, monitoring, and mandatory reporting systems^86^. Insights from the study help in on-ground execution of policy and enhanced SM adoption through proposed strategic measures (Fig. 4; Table 5) and strengthening enforcement by preventing inappropriate waste disposal practices (Table 3).

Practically, the findings call for more rigorous enforcement of CDW Management Rules, economic incentives such as a reduction in GST and subsidies, and improved communication strategies to increase awareness. The study underscores the need to decentralize recycling infrastructure, ensure just-in-time availability through local vendors, and mandate third-party certification to build trust. Additionally, targeted marketing, capacity building, and financial support for small-scale recyclers are critical for fostering a viable SM market in India. These combined actions can accelerate the transition to a circular economy and promote sustainable construction practices.

## Conclusions

As cities globally face escalating climate challenges, aligning with SDGs 9, 11, and 12 demands robust, localised, and sector-specific interventions. This study investigates the role of construction and demolition contractors in advancing a CE within the construction sector, specifically focusing on establishing a viable SM market for inert CDW in Chennai, India.

Using a mixed-methods approach, the study identifies critical gaps in awareness, compliance, and enforcement of local CDW regulations. Findings reveal low regulatory awareness and limited compliance among contractors, highlighting the need for targeted awareness campaigns, stronger monitoring mechanisms, and inclusive policy design that accounts for waste generators’ practices.

To build a sustainable SM market, the study proposes several regulatory and operational strategies for market establishment and facilitating industrial readiness. The study highlights that coordinated efforts spanning government, industry, and community stakeholders are vital to address consumer hesitancy, regulatory gaps, and economic barriers such as high taxation on recycled materials.

### Limitations

Although the sample size of 73 is adequate for exploratory research, it limits the statistical generalisability of the findings. Further, this study does not include scientific behavioural analysis of stakeholders and is geographically limited to the Indian context.

### Future Research

 Given the central role of client willingness identified in this study, future research should quantify clients’ willingness to pay for recycled materials and experimentally test the outcomes of proposed policy interventions (e.g., subsidies, tax breaks, penalties) and marketing strategies. Further research should also evaluate how improvements in clients’ purchasing behaviours translate into high material recovery and recycling rates to facilitate evidence-based policy design.

## Data Availability

The datasets used during the current study are available from the corresponding author on reasonable request.
